# Robotic Anesthesia – A Vision for the Future of Anesthesia

**Published:** 2011-10-17

**Authors:** Thomas M Hemmerling, Riccardo Taddei, Mohamad Wehbe, Joshua Morse, Shantale Cyr, Cedrick Zaouter

**Affiliations:** * Departments of Anesthesia, McGill University, Montreal, Canada University of Pisa, Pisa, Italy

**Keywords:** robot, anesthesia, closed loop, McSleepy

## Abstract

This narrative review describes a rationale for robotic anesthesia. It offers a first classification of robotic anesthesia by separating it into pharmacological robots and robots for aiding or replacing manual gestures. Developments in closed loop anesthesia are outlined. First attempts to perform manual tasks using robots are described. A critical analysis of the delayed development and introduction of robots in anesthesia is delivered.

The word robot was defined by the Josef Čapek, brother of the Czech writer Karel Čapek, who used it in the title of his play ‘Rossum’s Universal Robots in 1920. (1) In a factory that makes artificial people called robots are created who can think for themselves. There are several modern definitions of the term. The Encyclopaedia Britannica (2) defines it as “any automatically operated machine that replaces human effort, though it may not resemble human beings in appearance or perform functions in a humanlike manner”. Merriam-Webster (3) defines a robot as a “machine that looks like a human being and performs various complex acts (as walking or talking) of a human being”, or a “device that automatically performs complicated often repetitive tasks”, or a “mechanism guided by automatic controls”.

Robotic surgery is a reality, not a fiction. Surgeons have had the possibility to use commercial robots for more than a decade (FDA clearance for the DaVinci surgical robot in the year 2000). Anesthesiologists are confronted by an increasing number of surgeries performed with the aid of robots. The most commonly used surgical robot, the DaVinci surgical system is available in approximately 1000 hospitals in the US alone.

Surgeons came a long way from cutting open huge cavities with knives, very much like our ancient predecessors had done, to small incision surgery and robotic surgery within the short period of no more than 2 decades. And during all this development, we anesthesiologists have mostly used gas anesthesia not very different from the way Crawford Long used it in 1842.

Our specialty has a safety record like no other; despite the fact that its practitioners are surrounded by over 100 parameters in the operating room. The ‘pilots of the human biosphere’ (4) are left behind the technological revolution their brethren in aviation have enjoyed over the last decades.

Why has anesthesia not caught up with the development of surgery? Whilst our surgical colleagues are taking out prostrate glands in the 3D world of the DaVinci surgical system, sitting comfortably in a corner of the OR and moving remote robotic arms worthy of a science fiction film, most of us turn an antique knob of a volatile vapor. And most of us will not even use any objective control of the effects of these anesthetic gases, other than some empirical assessment.

Why has anesthesia not caught up with the technological development of surgery?

We will list some reasons and will try to explain them in more details: unused technology, concentration on safety in sheer survival, technological developments which did not keep their promises, concentration on safety and not enough on quality, emphasizing airway control rather than anesthesia control, and financial considerations.

## Technology available but not used

Twelve years ago, the corresponding author arrived in North America, confronted with an absence of bispectral index monitoring (BIS) devices. When the first BIS monitors and they were used, the predominant numbers which could be seen during maintenance were in the 20ies! It was clear that the predominant practice of anesthesia favored a too profound anesthesia with concomitant reduction of needs for opioids and muscle relaxants. This form of anesthesia leads to patients who start breathing more or less immediately after surgery but have a markedly reduced cognitive function and recover from anesthesia very slowly. Recent evidence indicates that too profound anesthesia might actually be harmful for our patients (5); exact titration of anesthesia, however, necessitates monitoring depth of consciousness. The predominantly used monitoring system, the bispectral index monitor, is not perfect but it is the best available monitor at present. Its use helps us to better assess the patient’s state of consciousness than relying on surrogate measures or empirical data. It is not and has never been designed to be an ‘awareness’ monitor, because awareness is a very complex problem whose exact mechanisms we have not fully understood yet. We are convinced from the evidence that any general anesthesia should mandate the use of some sort of depth of consciousness monitor.

## Safety is more than survival

We have long focused on establishing an excellent safety record, in fact one of the best of all medical specialties. This could be justified by the almost daily experience of our patients’ predominant concern, the risk of ‘not waking up’. However, other aspects define also the quality of anesthesia: the maintenance of hemodynamic and physiological control, the way how patients wake up, the recovery from anesthesia, the effect on memory, or the control of temperature. Maintenance of body temperature during surgery is still one of our main problems. Despite conclusive evidence of the harmful effect of failing to maintain body temperature during surgery, we still have not found a technologically satisfying solution to maintain body temperature throughout all types of surgery. So yes, we have an excellent safety record when we only look at how many patients die or suffer from life-threatening events due to anesthesia; but when we look more deeply, on performance overall, efficiency to maintain body temperature, stable blood pressure or heart rate, electrolyte balance, glucose control, there is room for improvement, and room for technological innovations.

## Target controlled infusion- a failed concept?

Target controlled infusion (TCI) pumps have been hailed more than 15 years ago as a big step forward in anesthesia. They are – simplified – microprocessor-controlled syringe infusion pumps which integrate pharmacokinetic models, based on studies of patient populations where virtual target concentrations were compared with ‘real’ concentrations in the blood in order to determine models which could be used to predict drug infusion rates. Early studies showed advantages over conventional types of anesthesia: some were merely based on the fact that TCI anesthesia is a total intravenous anesthesia, so patients naturally had less nausea or vomiting after surgery, some showed that wake up could be quicker because TCI pumps reduce the infusion rate of propofol over time, which most of us don’t. Overall, they have the potential to reduce our workload. However, without proper depth of anesthesia monitoring, models alone cannot predict individually titrated anesthesia. The fact that the amount of propofol necessary to maintain a certain level of anesthesia during surgery also relies on how analgesia is provided, something which these single-drug pumps do not take into account, makes their use rather more difficult than standard pumps: there is the risk that one relies too much on their predicted target concentration rather than on some interactive concept between analgesia and hypnosis: one wonders why there was never dual-TCI pump, e.g. integrating both remifentanil and propofol. So, TCI pumps are not a failure: they offer some interesting features, such as the reduction of propofol infusion rates over time and probably a better initial infusion rate, but should be used in conjunction with depth of consciousness monitoring systems.

## Inter-individual quality differences

The safety of our patients is a very important goal, but quality of anesthesia is also important. We underestimate the influence of human factors, similar to our surgical colleagues. It is not a myth that the quality of surgery depends on the skills of the individual surgeon. Surgical robots have helped to standardize certain surgical skills: the use of the DaVinci steadies the surgeon’s hands, therefore minimizing the influence of ‘shaking’. The importance of individual skill sets in anesthesia is equally known: an endotracheal intubation which looks easy and elegant in the hand of one anesthesiologist can look difficult, cumbersome or might even be impossible in the hands of another. Whereas the general public no longer accepts that their car does not function well because of the individual who built it, but expects equal quality in millions of cars, we accept huge inter-individual differences in the quality in the medical field. Robots have dramatically changed the industrial world; robots can do the same for anesthesia.

## From airway control to anesthesia control

Videolaryngoscopes have helped us to perform endotracheal intubation; laryngeal mask airways have replaced the need for endotracheal intubation in many cases. We need to develop smaller videolaryngoscopes, which can be used on an everyday basis, and we need to record the intubation process in all our patients so we can store this information for each patient: would it not be important to have a look at the previous intubation of our patient BEFORE we actually do it again? The way videolaryngoscopes have changed the way we establish airway control in our patients, robots can change the way anesthesia is delivered.

## Financial considerations

Anesthesiologists have problems getting all the technological advancements because we are not treated equally to surgeons. Whereas surgeons push very hard to get the latest devices, very often ignoring any cost issues, we have to fight to get BIS sensors for all our patients. When one watches the wasted sutures, endoscopic devices or in fact any device, one wonders why similar attitudes are not common in anesthesia? It is clear that we have to work on our self-confidence and on selecting leaders in our departments who are able to introduce the latest technologies into our daily practice.

## So what about robotic anesthesia? How can we achieve this?

Whereas surgical robots focus on aids or replacements for manual gestures, robots for anesthesia need to cover both, pharmacological applications and manual skills.

Therefore, we can distinguish anesthetic robots in robots which help calculate the appropriate dose of an anesthetic agent (***pharmacological robots***) and robots which replace or aid with manual gestures, such as the insertion of intravenous cannulas, endotracheal intubation or nerve blocks (***manual robots***).

As with most robots, the idea of robot design is to simulate human behavior but make it more reliable, more precise, void of emotions and tireless.

Therefore, robot design has to start with looking at what the human counterpart is doing and trying, specifically in the initial stages, to simulate this behavior.

## Pharmacologic robots

If we come back to the definition which we have given before, robots can be software programs automatically taking decisions for human beings. The best starting point for pharmacological robots is the principle of closed loop control.

Closed loop control of anesthesia means administering anesthetic agents through an actuator, in most cases a syringe infusion pump, determining the effect of these agents on the patient’s body by recording in real time parameters which are direct or if not available indirect surrogates of the effect we want to achieve with a given drug, e.g. muscle relaxation as measured using evoked twitch responses as a reflection of the action of muscle relaxants, using algorithms in some sort of ‘closed-loop brain’ and using this feedback information in clinically and pharmacologically sensible time intervals to adjust the next dose we will give.

A closed loop system changes the dose according to the information it gets from the patient, similar to an anesthesiologist in his or her everyday practice. An anesthetic robot should control all three components of anesthesia, hypnosis, analgesia and muscle relaxation, taking into account the complex interaction between the three components. Such a system is ultimately superior to simple TCI pumps, which do not close the loop, thus not determining the effect of the dose they administer and do not take into account all three components of anesthesia and their interactions with each other, something any good clinician would do.

## Closed loop systems

Closed loop systems have been tested for several components of anesthesia for more than two decades and they are at least as good as manual control, or better.

### Closed loop for hypnosis

The most widely researched closed loop system is for control of hypnosis; let’s call these closed loop systems *single-loop systems*, as they control only one component of anesthesia. A well researched system is the CLADS system (6) which uses feedback control from BIS to administer propofol. The ‘control algorithm’ of that system is based on the relation between various rates of propofol infusion and BIS based on standard pharmacokinetic variables. The system alters the propofol infusion rate based on the offset from target (BIS=50), the time elapsed since the initiation of infusion, the time delay factor between sensing and averaging of BIS data, the time delay between the change in infusion rate and the actual change in the plasma concentration of propofol and its peak effect. This system was tested in several patient populations, including open heart surgery. (7) Despite the small size of the study (22 patients in the CLADS group vs 22 patients in the manual group), the results indicate significantly better control of hypnosis and better outcome in terms of hemodynamic stability in the closed loop group: less propofol during induction, thus, less overshoots of BIS and less hypotension. Not only was the control of hypnosis as reflected in the Varvel parameters better, but this better control lead to less hypotension and less use of phenylephrine during surgery. We could show similar results using also a single-loop system. (8) In a randomized controlled trial on 40 patients undergoing major surgery, clinical and system control was significantly better when a closed loop system was used than with human manual control.

Dr. Liu’s research group has integrated control of both, remifentanil and propofol, via BIS monitoring. The degree of offset of the actual BIS from the target BIS is used to control remifentanil and propofol infusions: if the offset is small, the infusion rate of remifentanil is changed, if the offset if higher, the infusion rate of both drugs is changed. This dual system uses TCI pumps as the basis of their algorithms. Clinical research results show a better control of hypnosis than manually controlled hypnosis. This dual-loop system (dual in terms of control of two drugs, but single since it uses the BIS parameter for controlling both drugs) has been used in more than 1600 patients [personal communication Dr. Liu] with no adverse events. One of their latest reports (9) presents 160 patients randomized in either receiving anesthesia via the dual-loop system or via manual control. As in other studies, BIS target control is significantly better with the closed loop system, and there is a significantly shorter emergence from anesthesia, although a difference of 1 min is of no clinical significance. Since both drugs are controlled via BIS, there is also the difficulty to assess the control of analgesia in both groups. There is no difference of systolic blood pressure, heart rate or episodes of hypotension or ephedrine use between both groups, indicating similar control of analgesia in both groups.

### Closed loop of Analgesia

There were early attempts to alfentanil infusion rates using blood pressure control. (10, 11) Whereas these systems functioned well in a small number of volunteers of patients, early studies did not compare them with manual or human control. Therefore, there is only one study which compared separate control of analgesia by adjusting the infusion rate of remifentanil. (12) Pain control during general anesthesia is impaired by the inability to effectively communicate with the patient. However, indirect parameters, such as reactions of the autonomic nerve system, changes in heart rate or blood pressure, are indicators of a patient’s pain. They have been used to guide opioid treatment in daily routine since the beginning of anesthesia. The clinician adjusts analgesia according to heart rate and blood pressure based on his experience and also surgical variables, such as an estimation of the degree or presence of a surgical pain stimulus. Opioids are known to block changes in heart rate or blood pressure during periods of surgical stimuli. We have therefore combined these parameters into a novel pain score, called analgoscore, which gives, similar to a standard pain score system used in awake patients, an indication of the patient’s pain during surgery. The range of the analgoscore is defined from −9 (too profound analgesia) to 9 (insufficient analgesia) in increments of 1. To maintain a target of 0 is considered as the optimum balance between opioid administration and sufficient pain control for any patient under general anesthesia. In a recent study (12), when compared to manual control of analgesia, the single-loop system, delivered equal analgesic control.

Closed loop systems for analgesia are as feasible as closed loop systems for hypnosis. Possibly because of the difficulty of measuring pain directly and relying on surrogate measurements such as hemodynamic control, or new scores such as the analgoscore, since they are based on hemodynamic variables, and therefore too robust to show differences in pain control between machines and humans.

## Closed loop control of muscle relaxation

Closed loop systems for muscle relaxation have been used for research practices, e.g. when the impact of different anesthetics on the muscle relaxation was studied. (13) A study by Stadler et al. (14) developed an advanced controller for the application of mivacurium. The controller was able to maintain a preselected degree of muscle relaxation with excellent precision while minimizing drug administration. The controller performed at least equally well as the anesthesiologist. Since muscle relaxation is not as inherently important for all surgery types, e.g. most anesthesiologists would not use much muscle relaxation for surgery of the extremities; smart systems for muscle relaxation can easily be developed depending on the need for muscle relaxation according to special types of surgeries. Intermittent application of muscle relaxants seems to be sufficient since there is no study showing a better outcome when continuous and profound muscle relaxation is maintained throughout surgery.

## The principle of robotic anesthesia – McSleepy^™^

In order to create a true Anesthesia robot, all three components of anesthesia, hypnosis, analgesia and muscle relaxation need to be controlled automatically, from induction to emergence. ‘McSleepy’ (http://www.mcgill.ca/newsroom/news/item/?item_id=100263) integrates closed loop control of all 3 components of general anesthesia with a user interface where the anesthesiologist can choose between fully automated or semi-automated control (manual control of one or more components). Its setup on a touch screen starts with a communication with the user: the user needs to put in relevant patient data, such as weight, age, height, ASA classification, but also information such as the type of surgery, whether the patient takes specific drugs which might interact with the system control, e.g. beta-blocking agents, or any additional information concerning the pain control, e.g. concomitant neuraxial anesthesia. In fully automated mode, the system will then induce anesthesia using remifentanil, propofol and rocuronium or cisatracurium.

There are several safety features built in, such as a time interval between propofol injection and injection of the muscle relaxant with the option to click a button when mask ventilation is difficult: if clicked, the system will not allow injection of the muscle relaxant to avoid the failed ventilation, failed intubation situation. The induction follows the Bayes principle, the system will use the pharmacodynamic results to categorize the patient’s pharmacological ‘personality’ and adapt doses accordingly. Throughout maintenance, McSleepy provides automatically control of hypnosis, analgesia and muscle relaxation. However, it needs to ‘know’ at which stage surgery is, so there are buttons which need to be pressed, indicating ‘prepping’, incision, or 20 min to the end. This again is a safety feature: once pressed, the device will not give any muscle relaxant anymore and question any manual administration of a muscle relaxant. There is the possibility to manually override or exit the system at any time; however, a decision support system will question any human override which violates safety features built into the device. Preliminary results showed (15) that hypnosis and analgesia was significantly better controlled in 30 patients undergoing surgery when compared to 30 patients undergoing similar surgeries the same drugs controlled by human anesthesiologists.

## Anesthesia robots to aid or replace manual gestures

Recently, (16) fiberoptic intubation was assisted in two mannequin intubations using the DaVinci system. In a phantom study, the Davinci was used to insert a block needle into a nerve block phantom. The first robotic endotracheal intubation in humans was recently performed. (http://muhc.ca/newsroom/news/introducing-world%E2%80%99s-first-intubation-robot)

The Kepler Intubation System (KIS) consist of a joystick, which is manipulated with high precision by the operator; this joystick is linked via a carbon fiber robotic arm to a standard videolaryngoscope, which offers the advantage that the endotracheal tube can be attached to it on a special port. It displays a cross hair, which is placed over the vocal cords using the joystick after which the endotracheal tube can easily be pushed into the trachea by a non-trained person. Its functioning resembles the DaVinci where special hand operating devices can easily be used using two fingers to manipulate the robotic arms within the patient’s cavity. Future studies will show whether this system can be operated with the same precision as human can and many more technological developments are necessary to have this system ready for everyday use. However, the barrier of performing manual gestures in anesthesia with the aid of a robot has been pierced.

Robots have the potential to deliver manual gestures with higher precision, and without fatigue.

Robotic anesthesia is the future of anesthesia. Robots will invade medicine as much as they have invaded our industrial production. In fact, whenever high quality products, such as cars, need to be manufactured, robots are part of the workforce. They never tire; they perform without emotions and continuous precision.

Anesthesiologists need not fear this development but drive it; in the not so far future, an anesthesiologist will work hand in hand with robots to provide best patient care allowing equal quality and performance, any place, any time.

## Figures and Tables

**Figure 1:**
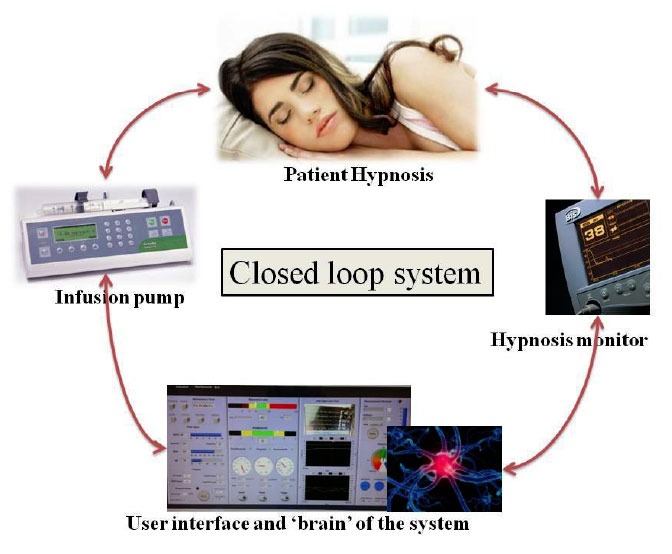
Illustration of a standard closed loop system for hypnosis; feedback control of hypnosis delivered by BIS monitoring

**Figure 2:**
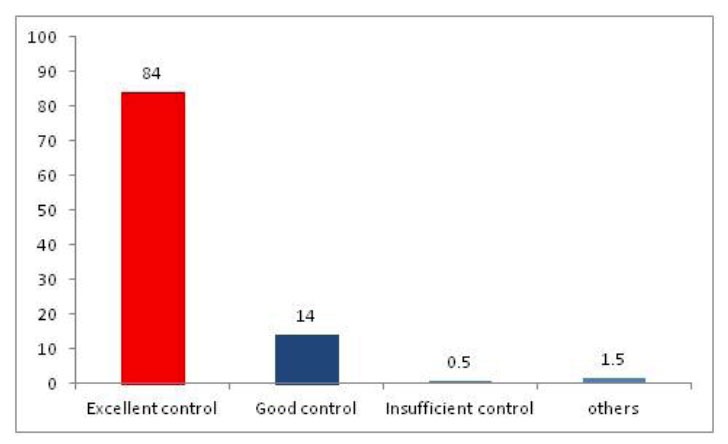
The figure shows the percentage of time during which different zones of analgesia control occurred for all surgeries (Excellent control represents an analgoscore between −3 and 3, good control represents analgoscore ranging from −6 to −3 and from 3 to 6 and insufficient control represents analgoscore ranging from −9 to −6 and from 6 to 9).

**Figure 3:**
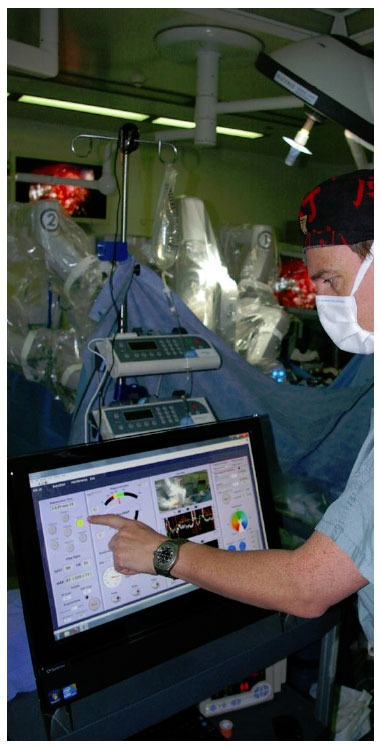
Illustration of touch screen interface of McSleepy^™^ during surgery

**Figure 4:**
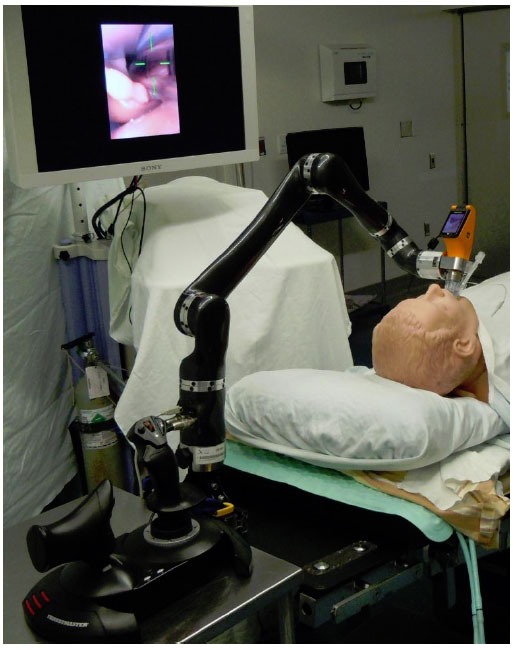
Illustration of the KIS: foreground: joystick with robotic arm; image shows cross hair within the vocal cords, endotracheal tube passing into the mannequin’s trachea
